# Supplementary material for: Study protocol for a randomized controlled trial: Effect of an everyday cognition training program on cognitive function, emotional state, frailty and functioning in older adults without cognitive impairment
Source: PLoS One. 2024 Mar 29;19(3):e0300898. doi: 10.1371/journal.pone.0300898 (PMC10980185; doi:10.1371/journal.pone.0300898)
Supplement: S2 File — (PDF) [file pone.0300898.s002.pdf]

The Research Ethics Committee of the University of Salamanca, in its ordinary meeting held on January 25, 2023, has considered the circumstances that concur in the research project entitled *"Effects of a training program in everyday cognition on cognitive function, emotional state, frailty and functionality in older adults without cognitive impairment: Randomized controlled clinical trial"*, whose principal investigator is Dr / a. Dr / a. Eduardo José Fernández Rodríguez.

In view of the documentation submitted, this Committee has agreed **to report favorably** the research project with registration number 902, since it meets the ethical requirements for its execution.

And for the record I sign it in Salamanca on January 26, 2023

GUTIERREZ RODILLA BERTA MARIA - 07843572C

Firmado digitalmente por GUTIERREZ RODILLA BERTA MARIA - 07843572C
Fecha: 2023.01.27 11:31:34 +01'00'

Fdo.: Luis Muñoz de la Pascua
Secretario del CBE

Fdo.: Berta Gutiérrez Rodilla
Presidente del CBE
