# Supplementary material for: Study protocol for a randomized controlled trial: Effect of an everyday cognition training program on cognitive function, emotional state, frailty and functioning in older adults without cognitive impairment
Source: PLoS One. 2024 Mar 29;19(3):e0300898. doi: 10.1371/journal.pone.0300898 (PMC10980185; doi:10.1371/journal.pone.0300898)
Supplement: S3 File — (PDF) [file pone.0300898.s003.pdf]

Application
Research project with human participants
and tissue samples (CEI-EP2)

Qualification: Effects of a daily cognition training program on cognitive function, emotional state, frailty, and functionality in older adults without cognitive impairment. Randomized controlled clinical trial.

☒ **New project** ☐ **Continuation** ☐ **Renewal of project no.** ☐ **Amendment**

1. Researcher:

Principal Investigator: EDUARDO JOSE FERNANDEZ RODRIGUEZ **Phone:**

Unit/Department: Department: Nursing and physiotherapy. **Fax:**

Address: Campus Miguel de Unamuno, C. Blood Donors, s/n, 37007 Salamanca **E-mail:**

2. Source of financing:

Organism: __________

State: ☐ Granted ☐ Required ☒ not subsidized

**Expected start date of the investigation (d/m/y):**	02/15/2023	☐ **in progress**
**Expected completion date (d/m/y):**	12/22/2023	

IP Compliance: The person who subscribes, as the researcher responsible for this project, informs: That he guarantees respect for dignity, free consent and the secrecy of personal data throughout the research process, declaring that he is responsible for compliance with the regulations applicable in the countries involved

IP Signature:

In Salamanca on FEBRUARY 15, 2023

Category proposed in the form (New project)

TO ☒ **Clinical experimental research with humans**
B: ☐ **Use of human tissues from patients or users, embryonic or fetal tissues Use of human tissues,**
C: ☐ **embryonic or fetal tissues, from sample or tissue banks Observational or experimental research**
D: ☐ **or use of personal data**

3. Personnel involved and qualification			
**List of people, including the IP, who will be in contact with the people participating in this study and the data or samples registered, as well as their category. If there is a student with no previous experience, describe her role and the supervision received (responsible person). All persons who participate directly in the process of obtaining data, their treatment, or have access to them, are subject to professional secrecy and comply with the requirements of the data protection law.** *(The space will be expanded if necessary)*			
**Name and surname**	**Category***	**Center**	
EDUARDO JOSE FERNANDEZ RODRIGUEZ Coordinator of the Occupational Therapy Degree. University of Salamanca. Faculty of Nursing and Physiotherapy. Cancer Area Researcher. Salamanca Biomedical Research Institute (IBSAL). Director of the doctoral thesis<br><br>SUSANA SÁEZ GUTIÉRREZ Doctoral thesis student program "Health, disability, dependencyand well-being".<br><br>CELIA SÁNCHEZ GÓMEZ PhD from the University of Salamanca. Associate professor. Dept. of Developmental Psychology and Education. University of Salamanca. Faculty of Psychology. Co-director of the doctoral thesis.			
*** PI, Collaborating researcher, technician, postdoctoral contract, predoctoral contract, other** *(specify)*:			

4. Description of the project *(the terms must be understood by people without training in the matter)*

4a. GENERAL OBJECTIVE AND BENEFIT: *(Describe the objective and general hypotheses and their potential benefit for human health and well-being, the advancement of scientific knowledge, or in the training of students)*

To evaluate the effectiveness of a daily cognition training program versus one of traditional cognitive stimulation in older adults without cognitive impairment, for cognitive function, emotional state, frailty, and functionality.

4b. SPECIFIC OBJECTIVES OF THE STUDY: *(Summarize the specific objectives structured in points)*

- To compare the differences in the emotional state of older adults without cognitive impairment between the daily cognition training program and traditional cognitive stimulation.
- To contrast the differences for the cognitive function of older adults without cognitive impairment between the daily cognition training program and traditional cognitive stimulation.
- To analyze the differences in the frailty index of older adults without cognitive impairment between the daily cognition training program and traditional cognitive stimulation.
- To identify the differences in the functionality of older adults without cognitive impairment between the daily cognition training program and traditional cognitive stimulation.
- To relate the interpersonal differences of older adults without cognitive deterioration with the results of scores in daily cognition, cognitive functions, emotional state, frailty and functionality.
- To correlate the results of the scores in everyday cognition with the cognitive function and instrumental activities of daily life of the individuals in the study sample.

4c. If it is a continuation of a previous project, indicate how the procedure differs:

4d. Indicate the section or subsection where it has been significantly modified *(only for modifications)*:

4e. KEY WORDS: use **<u>"ONLY" five keywords</u>**

"EVERYDAY COGNITION", "COGNITIVE FUNCTIONS", "EMOTIONAL STATE", "FRAGILITY", "FUNCTIONALITY"

5. Nature of the investigation

Research purpose*(check the most appropriate aspects)***:**

5a. Categories B and C of NEW PROJECT

1. ☐ **Research with human tissues**
   - 1.1. ☐ **Of patient-users**
   - 1.2. ☐ **From tissue banks**
2. ☐ **Research with embryonic tissues**
   - 2.1. ☐ **tissue banks**
   - 2.2. ☐ **Others**
3. ☐ **Other human biological samples** *(specify which one(s))***:**

5b. Categories A and D of NEW PROJECT

1. ☐ **Research with experimental methodology**
   - 1.1. ☐ **Electrical or magnetic stimulation and electrophysiological recordings (EEG, ECG, EMG, etc.)**
   - 1.2. ☐ **Acoustic or light stimulation and behavioral recordings (latency, accuracy of response, frequency, magnitude, etc.)**
   - 1.3. ☐ **Stimulation through visual or textual stimuli and behavioral registers (latency, accuracy, frequency, magnitude, etc.)**
   - 1.4. ☐ **Acoustic or light stimulation and electrophysiological recordings (EEG, ECG, EMG, etc.)**
   - 1.5. ☐ **Neuroimaging (IRfM, CT, etc.)**
2. ☒ **Research with correlational methodology and surveys (interviews, tests, questionnaires, scales, etc.)**
   - 2.1. ☐ **survey or interview**
   - 2.2. ☒ **Tests, questionnaires or psychometric scales**
3. ☐ **Research with observational methodology (scales or observation records, etc.)**
4. ☐ **Other kind of investigation not contemplated in the previous ones***(please specify which one)***:**

6. Fabric samples (Categories B and C) of NEW PROJECT

(Describe the nature of the samples)

7. Population and recruitment of participants or subjects (Categories A and D) NEW PROJECT
**7th Characteristics of the population (sample)**
1. ☒ **People WITH full capacity**<br>*The document Information Sheet for the Participant and Informed Consent for Research Projects in People with Full Capacity must be submitted (form CBE-A1, Privacy Policy, Informed Consent for People with Full Capacity, Revocation of Consent and, if there is a transfer of samples or data, Consent for Data Processing and Assignment of Image Rights, if any).*<br>**2. People WITH limited capacity to act**<br>☐ **2.1. people with age≤12 years**.<br>*The document Information Sheet for the Participant and Informed Consent for the guardian or legal representative must be submitted (form CBE-A2, Privacy Policy, Informed Consent in Research Projects in Minors or Legally Incapacitated Persons, and Revocation of Consent), Formal statement of the researcher / ay, if there is a transfer of samples or data, Consent for Data Processing and Transfer of Image Rights, if any.*<br>☐ **2.2. People aged > 12 ≤ 16 years.**<br>*The document Information Sheet for Minors and Informed Consent for the guardian or legal representative must be submitted (form CBE-A2, Privacy Policy, Informed Consent in Research Projects in Minors or Legally Incapacitated Persons, Revocation of Consent), Formal statement of the researcher /ay, if there is a transfer of samples or data, Consent for Data Processing and Transfer of Image Rights, if any.*<br>☐ **23. People aged > 14 years (scope of personal data).**<br>*The document Information Sheet for Minors and Informed Consent for the guardian or legal representative must be submitted (form CBE-A2, Privacy Policy, Informed Consent in Research Projects in Minors or Legally Incapacitated Persons, Revocation of Consent), Formal statement of the researcher /ay, if there is a transfer of samples or data, Consent for Data Processing and Transfer of Image Rights, if any.*<br>☐ **2.4. People aged > 16 ≤ 18 years (health field). mature minor.**<br>*The document Information Sheet for Minors and Informed Consent for the guardian or legal representative must be submitted (form CBE-A2, Privacy Policy, Informed Consent in Research Projects in Minors or Legally Incapacitated Persons, Revocation of Consent), Formal statement of the researcher /ay, if there is a transfer of samples or data, Consent for Data Processing and Transfer of Image Rights, if any.*<br>☐ **2.5. Permanent physical-psychic disability.**<br>*The document Information Sheet for the Participant and Informed Consent for the guardian or legal representative must be submitted (form CBE-A2, Privacy Policy, Informed Consent in Research Projects in Minors or Legally Incapacitated Persons, and Revocation of Consent), Formal statement of the researcher / ay, if there is a transfer of samples or data, Consent for Data Processing and Transfer of Image Rights, if any.*<br>☐ **2.6. temporary incapacitation**.<br>*The document Information Sheet for the Participant and Informed Consent for the guardian or legal representative must be submitted (form CBE-A2, Privacy Policy, Informed Consent in Research Projects in Minors or Legally Incapacitated Persons, and Revocation of Consent), Formal statement of the researcher / ay, if there is a transfer of samples or data, Consent for Data Processing and Transfer of Image Rights, if any.*<br>3. ☐ **Others***(specify which one(s))*:
**7b. Recruitment mode and participation criteria**

1. Recruitment means: ☒ **Direct contact (oral or written)** ☐ **Via Internet**

2. Participation criteria: ☒ **Voluntary**

Describe the mode of recruitment: The participants were recruited in the Occupational Therapy program of the Geriatric Revitalization project organized by the University of Salamanca in collaboration with the Hon. Salamanca City Hall.. In this recruitment phase, the participants voluntarily present their applications in the senior centers of the Salamanca city council. After acceptance into the program, it is determined which of them meet the inclusion criteria and none of the exclusion criteria, to be part of the study. There will be a presentation session, in which there will be a reading of the information sheet and the informed consent will be delivered for later signing if they agree to participate in the study.

8. Incentives or Measuring Instruments (Categories A and D) of NEW PROJECT

8a. Stimuli of a physical and psychological nature used in research with experimental methodology

Describe the stimuli you use for the investigation and the possible harm or harm to the participants.

1. ☐ **Physical stimuli:** ☐ **acoustic** ☐ **luminous** ☐ **electrical/magnetic** ☐ **Pressure**

☐ **Other physical or chemical stimuli not covered in this section***(specify its nature, intensity or dose and known effects)***:**

Stimulus dimension:

current: mA _____ **dB** _____

Frequency (Hz): ______

Duration: Milliseconds_______ **Seconds**_____ **Minutes**______ **Hours** _____

Stimulation effect: ☐ **Innocuous** ☐ **Inconvenience** ☐ **bearable pain**

☐ **other effects***(describe them)*:

2. ☐ **Psychological stimuli:**

☐ **Images or photographs**
☐ **Film or video images Verbal**
☐ **material**

Dimension of the stimuli:

Intensity: ☐ **Low** ☐ **Half** ☐ **high**

Valencia: ☐ **Positive** ☐ **neutral** ☐ **Negative**

Stimulation effect: ☐ **Neutral** ☐ **slight discomfort** ☐ **annoying but bearable** ☐ **Very annoying**

☐ **other effects** *(describe them)*:

2.1. If the stimuli used are part of a standardized base (figures, sounds, words, etc.), indicate the complete reference where all the information about their characteristics appears:

2.2. **If the stimuli are specifically designed for research and have a clear emotional impact, they must present enough information for evaluation.***(the class and characteristics of the stimuli must be specified)* **:**

8b. Measurement instruments used in research with survey or observational methodology

☒ **Interview, test, questionnaire or psychometric scales.**

1. If standardized material is used, indicate the complete reference where all the information on its characteristics appears: TEST FOR THE ASSESSMENT OF EVERYDAY COGNITION (PECC), MONTREAL COGNITIVE ASSESSMENT TEST (MoCA test), YESAVAGE GERIATRIC DEPRESSION SCALE, MEASURE OF FUNCTIONAL INDEPENDENCE (FIM) AND FRAGILITY INDEX

2. If the survey is designed specifically for research, it must present enough information for evaluation:

9. Record of responses

1. Registration system

☒ **1.1. In person:** **1.1.1.** ☒ **Individual**
1.1.2. ☐ **collective**

☐ **1.2. Non-face-to-face:** **1.2.1.** ☐ **Via Internet** **1.2.1.1.** ☐ **Web page**
1.2.2.2. ☐ **Email**
1.2.2. ☐ **Via postal mail**
1.3.3. ☐ **Others** *(specify which one(s))*:

2. Type of record

☒ **2.1. Personal data** *(specify in each case the type of registration)*:

☒ **2.1.1. Survey, interview or psychometric scale: initial interview**

☐ **2.1.2. Video**

☐ **2.1.3. Audio**

☐ **2.1.4. Photography**

☐ **2.1.5. Other personal data:**

☒ **2.2. Non-personal data** *(specify in each case the type of registration)*:

☐ **2.2.1. Physiological data:**

☐ **2.2.2. Behavioral data:**

☐ **2.2.3. Psychophysical scales:**

☒ **2.2.4. Other non-personal data: data referring to cognitive function, emotional state, fragility and functionality**

10. Design and procedure (Categories A and D of NEW PROJECT

10.1. Design:

Include independent and dependent variables, group formation (treatment and control), and measures (pre, post, and follow-up). Document the criteria used in the formation of groups (random or natural groups or criteria) and their relationship to the design. Also, describe whether the independent variable is manipulated or selected. In the clinic, highlight especially the way to proceed with the control group.

APPENDIX 1

10.2. Procedure:

Include explicit information on the number of sessions, sequence of presentation of stimuli or the measurement instruments of each session, and their duration. In the case of experiments, if there are stimulus differences between groups, their duration, intensity, and frequency, as well as the interval between the stimuli, should be described. It can include the instruments of presentation of stimuli and recording of responses.

APPENDIX 2

Is there deception about the procedure? ☐ **YEAH** ☒ **NO**

If there is deception, can you determine the decision to participate in the experiment? ☐ **YEAH** ☐ **NO**

Describe what strategy you follow to inform the participants of the objective of the investigation, the manipulation of the independent variables, the recorded data or the elimination of any misinterpretation, at the time of the completion of the experiment or the recording sessions:

At the beginning, the information sheet will be read to the participant and the informed consent will be delivered. At the end of the intervention, a personal report will be delivered with the individual results and relevant information from the study.

11. Design and procedure (Categories B and C) of NEW PROJECT

For research projects involving human biological samples only

12. Other clarifications that the research team considers important not included in the rest of the form. Attach surveys/interviews here if they are to be used

In the initial interview, data will be collected on:
- Age.
- Gender.
- Level of studies.
- Civil status.
- Main occupation.
- Medication intake and therapeutic adherence.
- Social supports.

APPENDIX 1:

STUDY DESIGN.

A controlled, longitudinal, prospective, randomized experimental clinical study will be carried out with simple assignment using a parallel scheme (1:1) to each of the two groups: experimental and control. A pre and post intervention evaluation will be carried out on all participants. The allocation, evaluation and intervention will take place simultaneously in both groups. To avoid performance bias, the participants will not know to which group they have been assigned and with it the treatment they will receive, so blinding of the study participants will be carried out. The main investigator is the same person who will carry out the intervention process, therefore it will be impossible to carry out a double-blind masking (of the investigators). For the evaluation process, external occupational therapists will be incorporated to evaluate and record the evaluations, previously trained and trained with the aim of avoiding subjective biases in the process, thus achieving uniformity with the results. They will not know the intervention group to which each group is assigned, so the clinical trial will have blind evaluation masked by a third party.

Yo. SITE

The study will be carried out in eleven centers for the elderly belonging to the Hon. Salamanca City Hall, Spain.

ii. SAMPLE SELECTION

STUDY POPULATION.

The target population to be studied will be people over 60 years of age without cognitive impairment who will voluntarily agree to participate in the Occupational Therapy project of the Geriatric Revitalization program organized by the University of Salamanca within the framework of the Active Aging Research Agreement with Preventive Physiotherapy -PReGe.

INCLUSION AND EXCLUSION CRITERIA.

INCLUSION:

- Be 60 years of age or older.
- Voluntarily sign the informed consent about the project, authorizing their participation.
- Carry out the initial assessment.
- Be admitted to the program from the beginning of it.

EXCLUSION:

- Not having knowledge of reading and writing or significant deficit in language comprehension.
- Institutionalized person.
- Present a clinical diagnosis of cognitive impairment.
- Participate in another cognitive stimulation program during the intervention process.

WITHDRAWAL:

- Leaving the program.

iii. RANDOMIZATION.

The purpose of randomization in this clinical trial is to avoid selection bias, balancing the groups in a homogeneous and unpredictable manner. The assignment of the two intervention alternatives that are part of our hypothesis will be carried out by randomization of the experimental and control groups in a parallel ratio of 1:1. To determine which of the eleven centers for the elderly will belong to each group, a simple random assignment of the centers will be carried out. Epidat 4.2 software will be used to allow randomization to be unpredictable and concealment of the randomization sequence.

iv. SAMPLE SIZE.

To obtain the sample size of this study, the Epidat 4.2 program was used. A unilateral approach has been assumed, establishing a confidence level of 95% and a statistical power of 80%. It has been decided to take equal sizes in the two groups (ie R=n2/n1) and the Yates correction will not be applied. To calculate the sample size, everyday cognition was selected as the main variable of the study. The current bibliography on the subject was reviewed and the study carried out by Fernández et al. in 2018, where the different results found in the main variable of a group that received training based on daily cognition were compared to another that received cognitive stimulation.

traditional. The results were as follows: the experimental group (population 1 that received training in everyday cognition) improved 2.57 points in the main variable, while in the control group 0.39 points (population 2 that received traditional cognitive stimulation). If a 20% loss of the expected population is established. The sample size at the end of the study will be 50 participants in the experimental group and 50 in the control group (n=99).

Table 1.*Justification of the sample size. Source: self made.*

P1	P2	LEVEL OF TRUST	CAN STATISTICAL	SIZE SAMPLE	P EXPECTED OF LOSSES	SAMPLE ADJUSTED TOTHE LOSSES
25.7 %	3.9 %	95%	80%	82 total<br>➢ *41 group experiment ntal*<br>➢ *41 group control*	twenty%	99<br>➢ *fifty cluster experimental*<br>➢ *49 cluster control*

The following formula has been applied:

$$n_1 = \frac{\left(z_{1-\alpha/2}\sqrt{(1+\phi)\overline{P}(1-\overline{P})} + z_{1-\beta}\sqrt{\phi P_1(1-P_1)+P_2(1-P_2)}\right)^2}{\phi(P_1-P_2)^2}\ ;\ n_2 = \phi n_1$$

SELECTION OF PARTICIPANTS.

The participants will be recruited in the Occupational Therapy program of the Geriatric Revitalization project in one of the eleven senior centers of the Hon. City Council of Salamanca, Spain who participate in said activity. All the participants who met the inclusion criteria and none of the exclusion criteria were included.

v. DATA COLLECT

An Excel database will be used to collect and store data, which will be created exclusively for this study and will include all the study variables described below.

saw. DESCRIPTION OF THE VARIABLES UNDER STUDY

INDEPENDENT VARIABLES

INTERVENTION A: Training in everyday cognition. This intervention will be carried out with the experimental group. Tasks will be carried out where the participants must make use of the cognitive functions during the development of the following AIVD: preparation of food, care of the house, use of transportation, shopping, use of the telephone, medication, financial management and access to information. and present.

INTERVENTION B: Traditional cognitive stimulation. This intervention will be carried out with the control group. Tasks focused on specific cognitive functions such as: attention, memory, executive functions, orientation, praxis, calculation, visual perception and reasoning will be carried out.

DEPENDENT VARIABLES

The variables will be analyzed at two different moments: pre-intervention and post-intervention.

- everyday cognition.
- Cognitive function.
- Emotional state.
- Fragility.
- functionality.
- instrumental activities of daily living

INTERVENING VARIABLES

They will be collected in the registration sheet during the initial anamnesis of the participants beforebeginning the intervention process.

- Age.
- Gender.
- Level of studies.
- Civil status.
- Main occupation.
- Medication intake and therapeutic adherence
- Social supports

vii. EVALUATION INSTRUMENTS AND DATA COLLECTION

A record sheet was made with the data of the clinical history and the results of the objective tests. Each of the participants will carry out an evaluation prior to the intervention process and another after it. These tests were carried out by occupational therapists qualified at the University of Salamanca and previously trained in the application of the tests.

DEPENDENT VARIABLES:

To evaluate the aforementioned variables, the following objective tests will be administered:

- **TEST FOR THE EVALUATION OF EVERYDAY COGNITION (PECC) (ANNEX 2)**

The Everyday Cognition Assessment Test measures the ability of older people to solve everyday problems through performance tasks whose resolution depends on their cognitive ability. The capacities analyzed are planning capacity and cognitive flexibility, verbal working memory, reasoning, episodic memory and crystallized intelligence. The result is a direct estimate of the person's functional capacity through 12 real situations grouped into 6 areas: medication, administrative management, financial management, food preparation, transportation, and shopping. Administration time is about 35 minutes.

- **MONTREAL COGNITIVE ASSESSMENT TEST (MoCA test) Version 8.3.**

It is a cognitive assessment tool created to detect mild cognitive impairment (MCI). Evaluates executive functions, attention, abstraction, memory, language, visual-constructive abilities, calculation and orientation. It lasts approximately 10 minutes to administer. The maximum score is 30 points, the cutoff for DCL being below 26 points (in developed countries).

- **YESAVAGE GERIATRIC DEPRESSION SCALE**

It is a depression screening questionnaire for people over 65 years of age. In this study, the 15-item version has been selected. It expresses the degree of satisfaction, quality of visa and feelings. Of the 15 items, 10 indicate the presence of depression if answered positively, while the remaining 5 answered negatively indicate this. Administration time is 5 minutes. A score above 5 shows moderate depression and above 10 severe depression.

- **FUNCTIONAL INDEPENDENCE MEASURE (FIM)**

It is a tool created to measure the level of functionality and assistance given by the caregiver. It assesses the activities of daily living subdivided into two dimensions: the motor dimension; that evaluates self-care (feeding, grooming, bathing, upper body clothing, lower body clothing, perineal hygiene), sphincter control (bladder and bowel), transfers (to and from the chair or wheelchair, transfer to the bathroom, bathtub or shower) and locomotion (walking or moving in a wheelchair, going up or down stairs). And the cognitive; which assesses communication (comprehension, expression) and social knowledge (social interaction, problem solving and memory). Each of the 18 items has a maximum score of 7 and a minimum of 1, with a total of 126.

- **FRAGILITY INDEX (FRAIL SCALE)**

It is an assessment designed to quantitatively measure frailty in geriatric patients. It consists of 5 questions about real situations in which a certain fragility can affect the quality of life. The maximum score of 5 points.

- **LAWTON AND BRODY SCALE**

It is an evaluation designed to assess autonomy in IADL in the elderly population. It is made up of eight items: telephone use, shopping, food preparation, housekeeping, laundry, transportation, medication control, use of transportation, and money. Its administration time is 4 minutes and 0 or 1 is valued in each of the items, with 8 for the total score. At present, it is influenced by a gender bias since it is more sensitive in women than in men (many of the activities have been carried out only by women throughout history).

INTERVENING VARIABLES:

In order to group and collect all the intervening variables, a clinical history was collected on an individual record sheet.

To measure therapeutic adherence, the **ARMS-e Questionnaire** which multidimensionally analyzes the lack of adherence in polypathological patients, allowing an individualized analysis of the barriers detected in adherence problems. It is a Linkert-type questionnaire in which there is no cut-off point, but the lower the score, the greater the adherence,with a total of 12 questions in which a value of 1 to 4 is assigned.

To measure social support, the **MOS Questionnaire,** since it allows us to quickly and easily evaluate social support in a global way, but it also allows us to know the emotional, instrumental, affective and positive social interaction dimensions. The first question of the questionnaire assesses quantitative social support and allows us to know the composition of the social network and whether it isfamily or friends. The following 19 questions are Likert type scoring from 1 to 5 qualitatively each of the dimensions mentioned above.

APPENDIX 2:

DESCRIPTION OF THE INTERVENTION:

PROCESS DESCRIPTION

The sequence of planning carried out in the study from October 2022 to May 2022 will be shown below:

1. PHASE 0: The recruitment phase will begin, in which the participants voluntarily present their applications in the centers for the elderly of the Hon. Salamanca City Hall, Spain. Being in mid-October when the lists of admitted to each of the eleven centers are published. After acceptance to the program, it is established who meets the inclusion criteria and none of the exclusion criteria (previously established).
   There will be a presentation session with each group of admitted, in which there will be a reading of the information sheet for the participant and informed consent will be given for their subsequent signature (if they agree to participate in the study).
2. PHASE 1:**Initial assessment,**in which each of the participants (control and experimental group) will be assessed by occupational therapists (previously trained in its application). For this, the aforementioned objective tests will be used and an interview will be held to collect the intervening variables (included in the registration sheet). All participants will be evaluated with the same tests. Subsequently, the simple random assignment of the study groups will be carried out.
3. PHASE 2: The**intervention**in which each of the eleven centers will have a weekly session, completing 25 sessions per center (275 total sessions).
4. PHASE 3: The**final assessment**, in which participants must complete the same objective tests as at the beginning. The results will be notified through a report individually and personally to the people who will request at the beginning the right to receive the information corresponding to the results and evolution of the process.

DESCRIPTION OF THE INTERVENTION

Two study groups were proposed that correspond to two interventions: one on everyday cognition training (experimental group) and another on traditional cognitive stimulation (control group). A simple random distribution of the eleven centers will be made. Each center will have a weekly session completing 25 sessions. The duration of each session will be 50 minutes. Being the first 10 minutes for the presentation of the session and the explanation of the activity, 30 minutes for carrying out the activity and the last 10 minutes for correcting the activities, farewell and final feedback. The work of the sessions will be individual, with the last part of the session being the one destined to share the results with the group.
He**control group**will carry out the program based on traditional cognitive stimulation. In this individual cognitive abilities such as attention, memory, executive functions, orientation, praxis, calculation, visual perception and reasoning will be trained. The materials and means used will be a cognitive stimulation notebook created for the study that includes printed paper sheets that participants must complete. Each day the homework will focus on training a specific cognitive function. Meanwhile **experimental group**
, will carry out a program based on everyday cognition, that is, on the use of cognitive functions to solve everyday and real problems that occur in our day to day and that allow us to be autonomous in our homes, such as preparing food, home care, use of transportation, shopping, use of the telephone, medication, financial management, and access to information and news. As material for use, as well as for traditional cognitive stimulation sessions, a daily cognition training notebook has been created that includes all the sessions mentioned above. Each day one of these tasks will be worked on.

Before beginning with the explanation of the typical session of our experimental group, it is important to explain the difference between traditional cognitive stimulation sessions and sessions based on everyday cognition training.

Traditional cognitive stimulation sessions aim to work on one or several cognitive domains in order to improve that specific cognitive area (Ortega et al, 2020). It is common to find studies that use "mental activation" notebooks such as the one carried out by Calatayud et al. in 2020 that use sets of cards in which isolated and specific cognitive aspects are worked on.
On the other hand, the sessions based on training in everyday cognition focus on the performance of tasks of daily life that are cognitively complex, but also cognitively demanding, that is, we need to face these tasks in our daily lives. These activities include context and significance, since they are problems or actions that the person must solve in their daily life in order to maintain their autonomy and independence. Currently there is little information in our country about training in everyday cognition, highlighting the study by Gómez and collaborators that introduce practical examples about it.

Table 3.*Planning of the sessions in the control group and in the experimental group.*

	Control group	experimental group
**Guy of intervention**	*stimulation cognitive traditional*	*Training in cognition everyday*
**Distribution of the sessions**	1.ATTENTION<br>2.MEMORY<br>3. ORIENTATION<br>4.PRAXIS<br>5.CALCULATION<br>6. ISUAL PERCEPTION<br>7. EXECUTIVE FUNCTIONS<br>8.REASONING	1.FOOD PREPARATION<br>2. HOUSE CARE.<br>3. USE OF TRANSPORTATION<br>4.PURCHASES<br>5. USE OF THE TELEPHONE<br>6. TAKING MEDICATION<br>7. FINANCIAL MANAGEMENTS AN ADMINISTRATIVE<br>8. ACCESS TO INFORMATION AND NEWS

viii. TYPE SESSION DESCRIPTION

Experimental group: Training in everyday cognition

One of the AIVDs carried out daily by older people who live independently in their homes is the*meal prep*. This is one of the key activities that creates a greater degree of dependency and significantly increases the use of care resources in this population, and that is why the daily cognition training program focuses on this activity.
For this, a 50-minute session has been planned in which a supermarket magazine will be used as complementary material. It is important that this magazine is updated at the time of its use and is from a supermarket that exists in the city where it is used (favoring temporal and spatial orientation).
The beginning of each session the person is asked to indicate the day, date andtime and each day they will be asked a different question in which they must use their memory to answer.
Participants will be asked to prepare a daily menu for three people with this magazine. But we will put one condition and that is that the budget cannot be less than 15 euros or more than 25 euros. The first step will be to select the ingredients, the second to number the dishes on the menu, and the third to describe the steps of the recipes that will be followed to prepare them. At the end we will share our menus and our recipes.

Another of the IADLs that generate dependency and health complications in the elderly is*taking medication.*In one of the sessions proposed for his training two tasks are requested, in the first one it is explained that Marisa has gone to the doctor for Sintrom and has been given a leaflet that she must read and understand to answer the questions correctly. In the second task she has to read, understand and memorize the medication sheet and answer some questions, but this time she will not be able to have the sheet in front of her when she answers the questions.
These sessions work on cognitive functions such as attention, retention, calculation, orientation, comprehension, visual, working and recent memory, among others. But there are also added benefits due to the context

generated by group dynamics that improve the emotional state of the participants. That is why the sessions should always keep in mind concepts such as the situation of equality, the feeling of belonging and unconditional acceptance. Avoiding any type of prejudice, moral connotations and feelings of isolation and inferiority.

Control group: Traditional cognitive stimulation

To end this section, we will highlight an example of the sessions in which specific cognitive functions such as attention will be worked on the activity begins with an explanation of why attentionis important in our day to day. Exercise 1 consists of describing everything we see in an image, and then answering some questions about it
