# Supplementary material for: Study protocol for a randomized controlled trial: Effect of an everyday cognition training program on cognitive function, emotional state, frailty and functioning in older adults without cognitive impairment
Source: PLoS One. 2024 Mar 29;19(3):e0300898. doi: 10.1371/journal.pone.0300898 (PMC10980185; doi:10.1371/journal.pone.0300898)
Supplement: S4 File — (PDF) [file pone.0300898.s004.pdf]

PARTICIPANT INFORMATION SHEET

RESEARCH PROJECT TITLE: "EFFECTS OF A EVERYDAY COGNITION TRAINING PROGRAM ON COGNITIVE FUNCTION, EMOTIONAL STATE, FRAGILITY AND FUNCTIONALITY IN ELDERLY ADULTS WITHOUT COGNITIVE IMPAIRMENT. RANDOMIZED CONTROLLED CLINICAL TRIAL".

IP: EDUARDO JOSÉ FERNÁNDEZ RODRÍGUEZ

INTRODUCTION

You have been invited to participate in a research study. Please take the time you need to read the following information and consult what you want. Please ask the researcher of this study if there is anything that is not clear to you or if you would like more information. This project has been favorably reported by the Research Ethics Committee of the University of Salamanca and follows the ethical recommendations of the Declaration of Helsinki.

You have been invited to participate in this research study because you belong to the Occupational Therapy program organized by the University of Salamanca and the Hon. Salamanca City Council within the framework of the Active Aging Research Agreement with Preventive Physiotherapy - PReGe.

THE PURPOSE OF THE STUDY

The main objective of this project is to determine the effects of a training program in everyday cognition for older people. Being everyday cognition the ability of people to solve problems that are complex in the everyday world and that allows us to carry out instrumental activities of daily life such as preparing food or managing money in a functional way. This helps us to be autonomous and to live in our homes, thus increasing our quality of life.

PROCEDURES

This document requests the collection of information on their cognitive, emotional and functional status. In addition to its evolution after the intervention process.

You will randomly actively participate in one of the two intervention programs that are the subject of the study, which are: a traditional cognitive stimulation program in which basic cognitive functions such as attention, memory, perception, etc., will be worked on through sheets, among others. And a daily cognition program in which, through different sessions, we will work on solving problems that you present on a day-to-day basis, such as preparing food, managing money or organizing administrative procedures. For each group there will be 25 weekly sessions of 50 minutes duration.

You will have to complete the initial and final evaluation with your personal data: name and surname, center for the elderly, age, gender, educational level, marital status, main occupation, social support assessed through the **MOS Questionnaire** and adherence to treatment through **ARMS-e Questionnaire**. All this data will be collected at the beginning of your participation.
You will also need to pass the following tests:**THE TEST FOR THE EVALUATION OF EVERYDAY COGNITION (PECC),** that will assess your everyday cognition,**THE YESAVAGE SCALE** that will assess your emotional state, **MONTREAL COGNITIVE ASSESSMENT TEST (MoCA test),** that will assess their cognitive performance and finally, **THE FUNCTIONAL INDEPENDENCE MEASURE (FIM),**who will appreciate its functionality, **THE FRAGILITY INDEX,** that quantitatively assesses its fragility and **THE LAWTON AND BRODY SCALE**, to assess their independence in instrumental activities of daily living.

PRINCIPLE OF NON-MALEFICENCE: RISKS AND DISCOMFORT

Participation in this study does not cause any discomfort, and does not imply any health risk.
The results obtained from your tests will be coded, filed and kept in accordance with current regulations at the Nursing Department facilities

and Physiotherapy from the University of Salamanca. All the information generated in this study will be used exclusively for the purposes specified herein.

TRANSFER OF DATA OR SAMPLES

In the event that your data is transferred to other research groups, it will always be done in accordance with current legislation, with your coded data, and to exclusively carry out studies related to the objectives of this work, and with prior authorization from the Ethics Committee of the Research the University of Salamanca. In the event that the objectives of the research work proposed by other research groups are different from those of this project, a new consent will be requested.

PRINCIPLE OF AUTONOMY AND BENEFITS OF YOUR PARTICIPATION: PARTICIPATION AND VOLUNTARY WITHDRAWAL

You are free to decide whether or not to take part in this study, participation is completely voluntary. If you decide to participate, you still have the possibility to withdraw at any time, without explanation, and without any penalty or negative consequences to you. If you change your mind about your samples or your data, you have the right to request its destruction, through the investigator. If you decide to participate, you must commit to doing to the best of your ability what the research team tells you.

RIGHT TO INFORMATION

There will be no economic benefits for participating in the study as it is an altruistic project, but you will be collaborating with occupational science and its evolution with the development of effective Occupational Therapy programs for the elderly.

At the end of the study, you will be able to consult the procedures, hypotheses, designs and overall results if they are available in accordance with article 27 of Law 14/2007 on Biomedical Research.

You will be able to choose if you want to obtain the research results that are relevant or not. The information will be sent to you through an individual report in which your results will appear in the initial and final assessment and where you will find information relevant to the intervention group that has participated. This report will be delivered to you in paper format at the end of the data collection in a personal and non-transferable way, under signature once it has been received.

CONFIDENTIALITY AND SECURITY MEASURES

All information used during this study will be treated strictly confidential in accordance with the privacy policy **(EXHIBIT)** and under the responsibility of the IP, in accordance with Organic Law 3/2018, of December 5, Protection of Personal Data and guarantee of digital rights and General Regulation (EU) 2016/679, of April 27, 2016, data protection and complementary regulations.

An effective anonymization system has been established that does not allow the subsequent identification of the participants. In no case will the consents granted, where the participants are identified, be combined with the questionnaires or other information used in the study. In the use made of the results of the study, for teaching, research and/or publication purposes, the proper anonymization of personal data will always be respected, so that the research participants will not be identified or identifiable.

If the results of the study are susceptible to publication in scientific journals, at no time will the personal data of the participants in this research be provided.

It is important that you do not discuss the characteristics of the procedures or the objectives of this study until all the research has been completed.

DATA AND CONTACT:

- NAME: EDUARDO JOSÉ FERNÁNDEZ RODRÍGUEZ
- EMAIL: edujfr@usal.es
- TELEPHONE NUMBER: 923294575

ANNEX: PRIVACY POLICY

Who processes your data?

The person responsible for the processing of your data is:

University of Salamanca CIF Q3718001E

C/ Patio de las Escuelas Menores, nº 1

CP 37008, Salamanca

How can you contact our data protection officer?

The data protection officer is the person in charge of supervising that we comply with the data protection regulations and helping you. If you have any questions or queries about how we treat the data, you can contact the data protection officer at: dpd@usal.es

Why do we process your data? Why and on what legal basis do we process your data?

We will process your data in order to manage your participation in the Research Project. Your data will be processed under:

Your consent (article 6.1.a) GDPR), to participate in the Project, and the publication of the results, where appropriate, in relation to the biographical references whose publication may be necessary in the Project.

Fulfillment of a mission carried out in the public interest or in the exercise of public powers conferred on the data controller (art. 6.1.e) GDPR) in accordance with the powers attributed to the University by virtue of articles 1 and 39 et seq. of the Organic Law 6/2001, of December 21, of Universities.

With whom do we share your data?

The data will only be communicated without the need to grant consent at the request of authorities.

In these cases, the University, before making the data available to third parties, ensures that these authorities request and access the data in accordance with the Laws.

How long will we keep the data?

The data will be used throughout the investigation until, where appropriate, the issuance of a report or the publication of its results.

The information will be kept duly blocked for the additional periods necessary for the prescription of possible legal responsibilities.

Information with historical value will be kept indefinitely with the prior approval of the Expurgation Commission by virtue of the provisions of Law 16/1985, of June 25, on Spanish Historical Heritage and the specific regulations applicable where appropriate.

How do we protect information?

As a public Administration, we apply the technical and organizational measures dictated by the National Security Scheme. This contemplates a series of recommendations to try to guarantee the security of information systems and thus avoid theft, alteration or unauthorized access to data.

In the case of subcontracting services, we will demand and ensure that the person in charge of the treatment applies measures similar to those of the National Security Scheme.

What rights do you have?

In order to maintain control over your data at all times, you have the right to access your personal information, as well as to request the rectification of inaccurate data or, where appropriate, request its cancellation or deletion. In certain circumstances, and for reasons related to your particular situation, you may oppose the processing of your data. Similarly, you can exercise the right to limit the processing of your personal information, requesting its conservation and also the portability of your data.

The exercise of rights is personal and therefore we need to identify you unequivocally. You can exercise your rights in two ways:

By sending an email message.

To do so, use this address: dpd@usal.es. We will only respond to requests made from email accounts provided by the University of Salamanca or that appear in our databases after identifying the owner.

By filing a document in our Registry or by postal mail addressed to: General Secretary

University of Salamanca. CIF Q3718001E

C/ Patio de las Escuelas Menores, nº 1

CP 37008, Salamanca

You must provide the following supporting documentation:

Proof of the identity of the interested party through any valid document, such as ID or passport.

Name and surname of the interested party or, where appropriate, of the person representing him, as well as the document accrediting such representation.

Petition in which the request is specified.

Address for notification purposes, date and signature of the applicant.

Supporting documents of the request that you formulate, if applicable.

In case of rectification or cancellation, indication of the data to be rectified or canceled and the reason that justifies it.

Who guarantees your rights? To whom can you complain?

In the event that you wish to file a claim or obtain additional information on the regulation of the processing of personal data in Spain, the competent authority is the Spanish Data Protection Agency (Jorge Juan, 6 28001-Madrid).

INFORMED CONSENT OF THE PARTICIPANT

PROJECT: "***Effects of a daily cognition training program on cognitive function, emotional state, frailty, and functionality in older adults without cognitive impairment. Randomized controlled clinical trial".***

I (Name, Surname and ID):________________________________I have been able to ask questions about the study.

I have received enough information about the study.

I have read the information sheet that has been given to me.

I have spoken with the Investigator:

NAME: EDUARDO JOSEÉ FERNÁNDEZ RODRÍGUEZ with DNI 70863702N I understand that my participation is voluntary.

I understand that I can withdraw from the study:

- 1º Whenever I want
- 2º Without having to give explanations
- 3º Without having any negative repercussions

I have also received information about the management of my personal data and its use, respecting current data protection regulations.

I voluntarily agree to participate in the project and authorize the use of all information obtained for it.

I understand that I will receive a signed copy of this informed consent.

CHOOSE ONE OF THESE OPTIONS ABOUT YOUR RIGHT TO RECEIVE INFORMATION:

☐Yes, I want to get the research results that are relevant to me. ☐I do not wish to receive information.

____

Signature of the participant

Place and date:

____

Name and signature of the researcher

Place and date

WITHDRAWAL OF CONSENT

I revoke the consent given on date   to participate in the project titled "____" and, for the record, I sign this revocation.

In___________, on___________of___________________of 20___.

Signature of the participant   Date:

Name and signature of the researcher   Date:
