# Supplementary material for: Study protocol for a randomized controlled trial: Effect of an everyday cognition training program on cognitive function, emotional state, frailty and functioning in older adults without cognitive impairment
Source: PLoS One. 2024 Mar 29;19(3):e0300898. doi: 10.1371/journal.pone.0300898 (PMC10980185; doi:10.1371/journal.pone.0300898)
Supplement: S5 File — (PDF) [file pone.0300898.s005.pdf]

ANNEX 1

MEDICATION TAKING SESSION

MEDICATION TAKING SESSION

DAY: __________ DATE: ______________ TIME: __________
QUESTION OF THE DAY : WHAT MADE YOU LAUGH YESTERDAY?

TASK 1: MARISA WENT TO THE DOCTOR FOR SINTROM. SHE HAS BEEN GIVEN THIS LEAFLET. READ IT CAREFULLY AND ANSWER THE FOLLOWING QUESTIONS.

KEEP THIS LEAFLET AS YOU MAY NEED TO READ IT AGAIN.
IF YOU HAVE ANY QUESTIONS, ASK YOUR DOCTOR OR PHARMACIST.
THIS MEDICINE HAS ONLY BEEN PRESCRIBED FOR YOU AND YOU MUST NOT GIVE IT TO OTHER PEOPLE, EVEN IF THEY HAVE THE SAME SYMPTOMS AS YOU, AS IT MAY HARM THEM.
IF YOU EXPERIENCE SIDE EFFECTS, CONSULT YOUR DOCTOR OR PHARMACIST, EVEN IF THEY DO NOT APPEAR IN THIS LEAFLET.

1. **WHAT SINTROM IS AND WHAT IT IS USED FOR**

SINTROM IS A MEDICINE THAT CONTAINS THE ACTIVE SUBSTANCE ACENOCOUMAROL. ACENOCOUMAROL BELONGS TO A GROUP OF MEDICINES CALLED ANTICOAGULANTS, WHICH REDUCE THE BLOOD'S ABILITY TO CLOT AND THEREFORE HELP TO PREVENT CLOTS FROM FORMING IN THE BLOOD VESSELS. SINTROM IS USED FOR THE PREVENTION AND TREATMENT OF BLOOD CLOTTING.

2. **WHAT YOU NEED TO KNOW BEFORE YOU START TAKING SINTROM**

YOU CAN ONLY TAKE SINTROM UNDER MEDICAL SUPERVISION. SINTROM IS NOT SUITABLE FOR ALL PATIENTS. DO NOT TAKE SINTROM
IF YOU ARE ALLERGIC TO ACENOCOUMAROL, OR TO ANY OF THE OTHER INGREDIENTS OF THIS MEDICINE LISTED IN SECTION 6.
IF YOU MAY HAVE PROBLEMS FOLLOWING THE TREATMENT (E.G. UNASSISTED SENILE PATIENTS, PATIENTS WITH ALCOHOLISM OR MENTAL DISORDERS).
IF YOU HAVE BLOOD DISORDERS WITH A BLEEDING TENDENCY OR SEVERE BLEEDING DISORDERS (E.G. HAEMOPHILIA).
IF YOU HAVE UNDERGONE OR ARE ABOUT TO UNDERGO MAJOR SURGERY SUCH AS EYE OR BRAIN SURGERY.
IF YOU HAVE UNDERGONE SURGERY (LUNG, PROSTATE, UTERUS, ETC.) INVOLVING HEAVY BLEEDING, INCLUDING DENTAL SURGERY, BECAUSE OF THE INCREASED CLOT-DISSOLVING CAPACITY OF THE MEDICINE.
IF YOU HAVE A STOMACH ULCER OR BLEEDING IN YOUR INTESTINES, BRAIN OR LUNGS.
IF YOU HAVE A SEVERE INFECTION OR INFLAMMATION IN YOUR HEART.
IF YOU HAVE UNCONTROLLED HIGH BLOOD PRESSURE.
IF YOU HAVE SEVERE LIVER OR KIDNEY DISEASE.
IF YOU ARE PREGNANT OR THINK YOU MAY BE PREGNANT.

PLEASE ANSWER THE FOLLOWING QUESTIONS (YOU CAN REFER TO THE LEAFLET ON THE PREVIOUS PAGE)

1.SHOULD YOU KEEP THE LEAFLET, AND WHY?

2. WHO SHOULD YOU CONTACT IF YOU HAVE ANY QUESTIONS?

3. WHAT IS SINTROM USED FOR?

4. IF YOU HAVE HAEMOPHILIA, SHOULD YOU TAKE SINTROM?

5. CAN YOU GIVE YOUR MEDICATION TO PEOPLE IN THE SAME SITUATION AS YOU?

TASK 2: NOW YOU ARE GOING TO READ CAREFULLY THE MEDICATION SHEET GIVEN TO MARISA, YOU WILL HAVE 3 MINUTES TO READ IT CALMLY, THEN YOU WILL HAVE TO ANSWER SOME QUESTIONS, BUT THIS TIME WITHOUT LOOKING AT THE PAPER. PREPARED? WHEN YOU ARE READY, TURN THE PAGE.

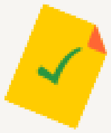

- **PLEASE ANSWER THE FOLLOWING QUESTIONS (THIS TIME YOU CANNOT REFER TO THE MEDICATION SHEET ON THE PREVIOUS PAGE)**

1. HOW MANY GRAMS OF SINTROM IS SHE PRESCRIBED IN HER TREATMENT?

2.WHICH OF THE FOLLOWING PATHOLOGIES HAS MARISA BEEN DIAGNOSED WITH? MARK THE CORRECT ANSWER.

HYPERTENSION, DIABETES, ARRHYTHMIA, ARTHRITIS, ARTHROSIS.

3.WHAT DAY WAS IT WHEN MARISA WENT TO THE DOCTOR, WHEN IS HER NEXT VISIT?

4.WHICH DAYS OF THE WEEK DOES MARISA HAVE TO TAKE A QUARTER OF THE PILL?

5.WHICH DAY SHOULD MARISA NOT TAKE ANY PILLS? WHY DO YOU THINK SHE IS NOT SCHEDULED TO TAKE ANY SINTROM ON THAT DAY?

MEDICATION SHEET

PATIENT'S DETAILS:
NAME: MARIA ISABEL GONZÁLEZ MARTÍN
AGE: 76
ACC. HAM/ TTO.: NO
TREATMENT: SINTROM 4MG
MÃRGENES TEST
DIAGNOSIS: ARRHYTHMIA
REMARKS: CLEXANE
START OF TREATMENT: 11/07/1997

SUMMARY OF THE LAST VISITS
DATE TEST
16/MARCH/2010
06/JAN/2010
04/JAN/2010
25/NOV/2009
15/NOC/2009
MEDICAL :
INTERNATIONAL NORMALIZED RATIO
INTERNATIONAL NORMALIZED RATIO
INTERNATIONAL NORMALIZED RATIO
INTERNATIONAL NORMALIZED RATIO
INTERNATIONAL NORMALISED RATIO

DETAILS OF THE VISIT
DATE: 15/03/2010
ACC. HEME / TREATMENT : NO
TEST :INR SINTROM
DRUG : SINTROM 4 MG (ACENOCOUMARIN)
DOSE : 11,00
PATTERN : 1/2 DAY - TUESDAY, THURSDAY,SATURDAY 1/4 DOSAGE

MONDAY	TUESDAY	WEDNESDAY	THURSDAY	FRIDAY	SATURDAY	SUNDAY
	16 mar 1/4 Comp	17 mar 1/2 Comp	18 mar 1/4 Comp	19 mar 1/2 Comp	20 mar 1/4 Comp	21 mar 1/2 Comp
22 mar 1/2 Comp	23 mar 1/4 Comp	24 mar 1/2 Comp	25 mar 1/4 Comp	26 mar 1/2 Comp	27 mar 1/4 Comp	28 mar 1/2 Comp
29 mar 1/2 Comp	30 mar 1/4 Comp	31 mar 1/2 Comp	01 abr 1/4 Comp	02 abr 1/2 Comp	03 abr 1/4 Comp	04 abr 1/2 Comp
05 abr 1/2 Comp	06 abr 1/4 Comp	07 abr 1/2 Comp	08 abr 1/4 Comp	09 abr 1/2 Comp	10 abr 1/4 Comp	11 abr 1/2 Comp
12 abr 1/2 Comp	13 abr 1/4 Comp	14 abr 1/2 Comp	15 abr 1/4 Comp	16 abr 1/2 Comp	17 abr 1/4 Comp	18 abr 1/2 Comp
19 abr 1/2 Comp	20 abr X 1/4 Comp					

ANNEX 2

ATTENTION SESSION

DAY: __________ DATE: _____________ TIME: __________

QUESTION OF THE DAY : WHAT DID YOU EAT YESTERDAY?

ATTENTION

Attention allows us to be prepared to respond to different situations that arise in our daily lives. It allows us to prioritise in the face of different external and internal stimuli, selecting what is important from what is not. This selection process prevents our brain from being overloaded due to the large amount of information we receive every day.

Would you like to start working on your attention?

EXERCISE 1:

LOOK AT THE PICTURE AND WRITE WHAT YOU SEE IN IT

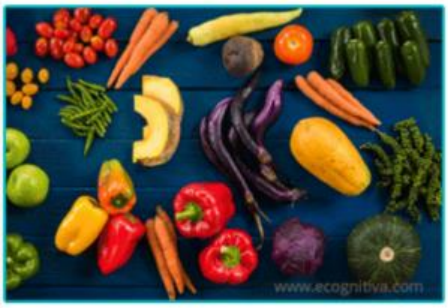

WITHOUT LOOKING AT THE PICTURE TELL ME IF...

- **WERE THERE FRUITS AND VEGETABLES?**
- **HOW MANY DIFFERENT FRUITS AND VEGETABLES WERE THERE? NAME THEM**
- **LIST AND QUOTE THE COLOURS THAT APPEAR IN THE PICTURE**
- **HOW MANY APPLES ARE THERE?**
- **ARE THERE 10 CARROTS?**
