# Supplementary material for: Study protocol for a randomized controlled trial: Effect of an everyday cognition training program on cognitive function, emotional state, frailty and functioning in older adults without cognitive impairment
Source: PLoS One. 2024 Mar 29;19(3):e0300898. doi: 10.1371/journal.pone.0300898 (PMC10980185; doi:10.1371/journal.pone.0300898)
Supplement: Attachment — Submitted filename: Response to Reviewers.docx [file pone.0300898.s008.docx]

Dear reviewer 3.

Firstly, I would like to express my gratitude for your valuable revisions. We hope that you will find them sufficient for publication. Please do not hesitate to contact us if you have any questions or if you require further modifications.

We believe that the manuscript has improved in quality thanks to your input, which will facilitate its continuation once it is put into action.

We will now attach all the responses and modifications we have made.

Sincerily,

Susana Sáez Gutiérrez.

**AUTHORS’ RESPONSE REVIEWER 3**

| **COMMENTS FROM REVIEWER 3** | Authors’ Responses |
| --- | --- |
| Introduction: It is still rather long and unclear. The information about the aim of the Occupational Therapy etc is not needed. The issue here is that not only the novelty of the study is lost (which is not relevant to this Journal's publication criteria) but the methods 'justification' in what each measure is doing, is lost. | Thank you for your comment. We agree with your evaluation of the changes in the introduction.  Therefore, we have thoroughly revised it to reduce its length and provide greater significance to the topic.  The following sentence has been removed:  **“and factors such as the presence of enabling environments and support can help older people to maintain their autonomy”**.  The following sentence has been replaced: **“Occupational therapy is a crucial healthcare science in providing functional maintenance to elderly individuals facing cognitive decline. This is achieved through the therapeutic use of daily life activities, which aim to encourage greater participation among the elderly both at home and within the wider community (12,13). Various approaches exist within this discipline to achieve this objective”.**  For the following:  **“Occupational therapy is one of the healthcare disciplines that use neurocognitive approaches to maintain or improve functionality in the elderly population” (12,13)** |
| Methods: 1. Remove the duplicate 'In order to ensure...' were established'.  2. Given this is a protocol paper, I think the methods should be crystal clear. Please clarify the below: '..To calculate the sample size, everyday cognition was selected as the main study variable..' - is that the PECC? '..improved 2.57 points on the main variable, while the control group improved 0.39 points' - is that points from a questionnaire, e.g. PECC? An expected effect size would be more useful to replicate the calculations -a rough estimation dictates you are expecting a moderate effect, is that correct? There seems to be some confusion on the 'qualitative' data and I am at least partially to blame for this. My understanding from the original submission was that there was some interview/focus groups qualitative data, hence my suggestion for thematic analysis. It appears that none of the primary or secondary outcomes includes an interview, so there are only quantitative data with possibly different levels of data as their outcome. Therefore, the thematic analysis comment as well as any reference to qualitative data should be removed. Further, the analysis appears to 'mix and match', while also making assumptions about the data - how do you know, for example, that some data will be normally distributed and others not? Even more so, the variables expected to be normally distributed appear to also be ordinal in nature. I think this needs further revisiting, in order to: a) consider the variables you have, why they were collected in the first instance and what they will tell you b) identify what level of data you have for those variables c) how you will analyse them and why; include any assumptions made or 'if x happens, we will do y' d) consider the tests to achieve that At the moment, there seems to be a somewhat random mix of approaches and analyses, with little justification. | Thank you very much for your comments and recommendations.   1. The run-on sentence has been deleted: **“In order to ensure the greatest fairness among the different groups, a maximum of 15 and a minimum of 5 participants per group were established”.** 2. The sample size has been calculated taking into account everyday cognition as the main variable, as you correctly stated. This was done by considering another similar study that also takes this variable as the main one. In both studies, a control group that undergoes traditional cognitive stimulation sessions is compared to an experimental group that undergoes sessions based on everyday cognition. The experimental group showed an improvement of 2.57 points compared to the control group's 0.39. In our study, we have chosen the PECC as the standardized test to measure the variable of everyday cognition.   We believe there may have been an error in the initial draft of the article we submitted. The data we will consider as the main dependent variables in the study are quantitative data derived from standardized tests. The only qualitative data, collected through an initial interview in the initial assessment of the participants, are the sociodemographic factors of the participants that will be analyzed as intervening variables. Therefore, we have removed the thematic analysis section as recommended and the following qualitative data section:  **“Qualitative variables in the study will be defined by frequencies and percentages. A thematic analysis will be conducted to identify key patterns and themes within the qualitative data, such as the variable "occupation", collected through an interview”.**  Regarding the raised question about the study variables, please find below the section of the article where the variables are explained: **“The primary variable will be everyday cognition, measured by the Test for the Evaluation of Everyday Cognition (PECC) and cognitive performance, measured by the Montreal Cognitive Assessment Test (MoCA). Secondary variables will be functionality (Functional Independence Measure, FIM), emotional state (Yesavage Scale), frailty (Frailty Index) and independence in instrumental activities of daily living (Lawton and Brody Scale). We will also record the following intervening variables in the clinical history of each participant: social support (Questionnaire MOS) and adherence to treatment (Questionnaire ARMS-e), and other socio-demographic data (age, sex, level of education, marital status and main occupation)”.**  It is true that we may have made an erroneous assumption that the distribution will be normal for some variables.  Therefore, the following sentence has been removed:  **“Sociodemographic variables and reference data, such as therapeutic compliance or level of social support, which have a normal distribution, will be defined by the mean and standard deviation, while variables which do not follow a normal distribution will be defined by the median and interquartile range”.**  Being replaced by the following:  **“Variables that have a normal distribution shall be defined by the mean and standard deviation, while variables that do not follow a normal distribution shall be defined by the median and the interquartile range”.** |
